# Supplementary material for: The experiences of family caregivers of people with severe mental illness in the Middle East: A systematic review and meta-synthesis of qualitative data
Source: PLoS One. 2021 Jul 9;16(7):e0254351. doi: 10.1371/journal.pone.0254351 (PMC8270161; doi:10.1371/journal.pone.0254351)
Supplement: S2 File — Showing full list of search terms that were applied in all databases. (PDF) [file pone.0254351.s002.pdf]

S1 Detailed Search Terms

The academic database search conducted using a list of predetermined search terms which were derived from four main search terms “family caregivers”, "experiences", “mental illness”, and “’Middle East”. These main search terms were further elaborated to include alternative terms. The search terms were applied across sources using advanced search option, the PICO structure and MeSH terms combined using Boolean operators.

(Family caregiver* OR; sibling* OR; parents OR; relative* OR; family member*) AND (Severe OR; mental illness OR; mental health OR; mental disorder OR; psychiatric disorder OR; psychosis OR; schizophrenia OR; bipolar) AND (Experience* OR; psychological distress OR; distress OR; depression OR; anxiety OR; stress OR; burden) AND (Middle East OR; Gulf Peninsula OR; Gulf countr* OR; Persian Gulf OR; Arabian Peninsula).
