# Supplementary material for: The experiences of family caregivers of people with severe mental illness in the Middle East: A systematic review and meta-synthesis of qualitative data
Source: PLoS One. 2021 Jul 9;16(7):e0254351. doi: 10.1371/journal.pone.0254351 (PMC8270161; doi:10.1371/journal.pone.0254351)
Supplement: Attachment — Submitted filename: Aisha-Response letter.docx [file pone.0254351.s003.docx]

**UCL Division of Psychiatry**

Aisha Hamed Alyafei

PhD student

UCL Division of Psychiatry

149 Tottenham Court Road

London, W1T 7NF, UK

Prof. Perla Werner

Academic Editor, PLOS ONE

3^rd^ June 2021

Dear Prof. Perla Werner

Thank you for your email dated 12^th^ April 2021 enclosing the reviewers’ comments. We would like to thank you and the reviewers for reviewing our manuscript **The experiences of family caregivers of people with severe mental illness in the Middle East: A systematic review and meta-synthesis of qualitative data.** Your comments have been very valuable and insightful, and we agree have led to improvements in the paper.

We have carefully reviewed each comment and have revised the manuscript accordingly. Our responses are given in a point-by-point manner in the table below.

| **Comments from Reviewer 1** | | |
| --- | --- | --- |
| **Reviewer 1** | **Our Response** | **Page** |
| **Title**  1. I recommend clarifying in the title that the systematic review is of qualitative data. the authors may consider the title "The experiences of family caregivers of people with severe mental illness in the Middle East: A systematic review and meta-synthesis of qualitative data". | Thank you for your recommendation, we have modified the title. | P1 |
| **Abstract** 1. In the conclusions, what do you mean by "Family caregivers experienced distress and burden which had a significant impact on their psychological well-being"? I think this sentence needs to be rewritten, it has not been directly examined and it is problematic to talk about a significant impact in this manuscript. | We have re-written the sentence and revised the Abstract sections: Methods, Results, and Conclusion. | P2-3 |
| **Introduction** 1. When authors present data or refer to conclusions from previous studies they should include citations. For example, see lines 54-56, page 4 (suggestion for citation: World Health Organization (2019). Mental disorders. Retrieved from: https://www.who.int/news-room/fact-sheets/detail/mental-disorders). Also see, lines 61 and 66, page 4, and line 90, page 5 (suggestion for citation: Abo-Rass, F., et al., (2020). Depression Illness Representations Among Arabs in Israel: A Qualitative Study Comparing Younger and Older Adults. Journal of Cross-Cultural Gerontology, 35(4), 353-366.‏ and Abu-Kaf, S., & Braun-Lewensohn, O. (2015). Paths to depression among two different cultural contexts: Comparing Bedouin Arab and Jewish students. Journal of Cross-Cultural Psychology, 46, 612-630). | Thank you for providing these references. After careful reading and consideration, we felt that these weren’t the most appropriate references to support our argument, however, we have now included other relevant citations to support our argument line 54-56 “Mental Illness [Internet]. The National Institute of Mental Health. 2021 Jan. Available from: <https://www.nimh.nih.gov/health/statistics/mental-illness.shtml>”; also lines 61-63 we included two citations “*Khalaila R. Caregiver burden and compassion fatigue among Arab family caregivers of older relatives. Journal of Applied Gerontology. 2020; 1-9. doi:10.1177/0733464820920100*; and *Yin M, Li Z, Zhou C. Experience of stigma among family members of people with severe mental illness: A qualitative systematic review. International Journal of Mental Health Nursing. 2019;29(2): 141-160. doi:10.1111/inm.12668*”; and line 123 we included another citation that support the paragraph “*Daher‑Nashif S, Hammad S H, Kane T, Al‑Wattary N. Islam and mental disorders of the older adults: Religious text, belief system and caregiving practices. Journal of Religion and Health, 2020;60: 1-15. doi:10.1007/s10943-020-01094-5”.* | P4 line 54-56  P4 line 61-63  P6 line 123 |
| 2. Paragraph 2, page 4 - The consequences of caring for a family member with MSI should be expanded. | We have now expanded on paragraph 2 and included new citations. | P4-5, line 61-86 |
| 3. The ideas of paragraphs 2 and 3 on page 4 (not the content) should be connected so the general idea will be clearer. You might start paragraph 3 with something as: unlike the western countries, in the Middle East, very little research exists regarding ... that is despite ... | Thank you for your suggestion. We have added some sentences at the end of paragraph 2 to connect the ideas in this section more clearly. | P5 line 82-86 |
| 4. In lines 83-84, page 5, it is mentioned "Few studies have explored in depth the experiences of caregivers in this region using qualitative methodologies", these studies should be presented, also there's findings and conclusions. (suggestions for this, see: Abojabel, H., & Werner, P. (2019). Exploring family stigma among caregivers of persons with Alzheimer's disease: The experiences of Israeli-Arab caregivers. Dementia, 18(1), 391-408). | Thank you for suggesting this reference. We have modified this paragraph and outlined three studies. | P6 lines 109-118. |
| 5. Lines 92-93, page 5, "Whilst Middle Eastern countries can be different from the West there is also great variation within different Middle Eastern countries". Which variation do you mean? The authors should clarify this sentence to avoid confusion | In response to the reviewer’s question, what we mean by variation is that there are cultural, economic, religious and language differences within different Middle Eastern countries. We have made some changes to paragraph 6. We have also explained what we mean by variations and listed few examples. | P7 line 129-134 |
| 6. Literature should be added regarding the status/importance of the family among individuals in the Middle East, and in Arab and Muslim culture. | We have expanded on paragraph 3 to include some literature about the status/importance of family in the Middle Eastern Muslim cultures. | P5-6, line 94-106 |
| 7. Lines 71-75, page 4; as there are several definitions for countries included in the Middle East, please clarify the source of the Middle East definition and add a citation. | We have provided a citation and a source of the Middle East definition. | P5 line 87 |
| **Method** 1. I suggest merging the sections "design" and "search strategy". In addition, the rationale of the systematic methodology should appear in the introduction. Please make sure there is no repetition. | Thank you for your suggestion. After careful consideration and discussion, we have decided to retain the two separate sections on design and search strategy as we felt it was helpful to distinguish the study design from the specific search strategy as it provides a logical structure and clarity for the reader. We have edited these sections to ensure there is not repetition.  In response to the point about providing a rationale for the systematic review, we have added a sentence explaining why systematic methodology is helpful in the introduction. | P8 Design/Search strategy.  P7 line 141-144 |
| 2. The section "selection criteria" should be focused and shortened. For example: no need for criteria No.4 in the excluded criteria. | In response to reviewer’s suggestion, we have shortened the “selection criteria” section (exclusion paragraph) from a total of 5 points to 4 points. | P9 line 189-194 |
| 3. In the section "selection criteria", inclusion criteria No.7 is not clear. Does it include major depression, dementia and Alzheimer? I am familiar with other studies that have qualitatively examined the experience of family caregivers for individuals with dementia in the Middle East. This criterion must be redefined. | In response to reviewer’s feedback, we have reworded the inclusion criteria No.7 as we are including schizophrenia, bipolar disorder according to DSMI-V or equivalent like Schizoaffective disorder.  In exclusion criteria No.4 line 191-194 we have made it clear that we are excluding non-SMI studies (caregivers of people with intellectual disability, developmental disorders, autism, dementia etc.) | P9 line 191-194 |
| **Results** 1. Regarding the "Themes" section; the section is very long; it should be reduced to fewer themes. For example, it is not clear how the sub-themes lack of patient's insight into the illness is related? However, you may combine it with the sub-theme lack of knowledge. Similar to this, the sub-themes "Restrictions", "Sole Responsibility" and " Self-Sacrifice" might be combined. I recommend rearranging and rewriting the themes. I also suggest reducing the number of quotes per theme and choosing only the most appropriate one. | Thank you for this feedback. We have modified and rearranged the Results “Themes” section and reduced number of quotes per theme.  In response to reviewer’s suggestion of combining the sub-themes, the lack of patient's insight into the illness was combined with the sub-theme lack of knowledge. Also, sub-themes: “Restrictions”, “Sole Responsibility” and “Self-Sacrifice” have not been combined under one subtheme “Self-sacrifice and responsibility”. | P20-32 |
| 2. In the "themes" section, as table 3 shows the "Overarching themes emerging from meta-synthesis", no need for figure 2. | Thank you for your comment. After consideration and discussion with the research team we have decided to retain the figure because we think that it adds further to the table as it has a visual depiction of the overarching themes and their inter-relationship. | P19 |
| **Discussion** 1. The authors could better explain and integrate the findings with existing literature. | We have revised the “Discussion” section and made changes to better integrate our findings with previous literature. | P32-35 |
| **Strengths and limitations** 1. In order to avoid repetition, it is recommended to focus only on the limitations of the review. the strengths and limitations of the included studies have already been presented throughout the article. | We have deleted the strengths and limitations of the included studies section. | P35 |
| **Implication for future research and practice** 1. In line 767, page 34, what different qualitative research do you mean? Please detail it. | Thank you for pointing this out. We have re-written the sentence as follows “To further enrich our understanding of family caregivers’ needs and experiences, we recommend conducting further in-depth qualitative research”. | P36 line 784 |
| The language of the current manuscript is unclear, making it difficult to follow. I advise the authors to improve the flow and readability of the text. | Thank you for your advice. The manuscript has been edited by an native English speaker, so we hope it is clearer now. | - |
| **Comments from Reviewer 2** | | |
| **Reviewer 2** | **Our Response** | **Page** |
| This is a rigorous and well-written review of an area of literature that has not been previously addressed in a review article. | Thank you for your comment. | - |
| It could be made more clear in the title that the review only focuses on qualitative studies, as this wasn’t evident to me until I started reading the body of the article. | Thank you for your recommendation, we have modified the title to make it much clearer that the review is of qualitative research. | P1 |
| An additional question I have is whether the focus on the Middle East is purely geographic, or if the focus is specifically on Muslim families within the Middle East. There have been a couple of qualitative studies conducted in Israel that included family members (Amsalem, D., Hasson-Ohayon, I., Gothelf, D., & Roe, D. (2018). Subtle ways of stigmatization among professionals: The subjective experience of consumers and their family members. Psychiatric Rehabilitation Journal, 41(3), 163-168.doi:10.1037/prj0000310; Amsalem, D., Hasson-Ohayon, I., Gothelf, D., & Roe, D. (2018). How do patients with schizophrenia and their families learn about the diagnosis? Psychiatry (New York),81(3),283-287. doi:10.1080/0033274 7.2018.1443676) that may have been excluded as they did not focus on Muslim families, but this should at least be clarified, as they otherwise appear to fit within the inclusion criteria. | In response to the reviewer’s question, the focus on caregivers in the Middle East is purely geographic, as studies included in this review did not specify whether they recruited Muslims or other religions within the Middle East.  Thank you for sharing these studies. We re-ran our searches, however, because of the search terms used, the suggested studies by the second reviewer have not been picked up and did not appear during indexing. The new searches did pick up two papers, so we have taken the opportunity to include these in the review and have updated the new figures from the later search date and have adapted our PRISMA flow diagram and text accordingly.  In terms of including the suggested studies in the review, one of them is pilot study, and after consulting the author Amsalem, he stated that both used the same cohort, therefore, we excluded this paper “*Amsalem, D., Hasson-Ohayon, I., Gothelf, D., & Roe, D. (2018). How do patients with schizophrenia and their families learn about the diagnosis? Psychiatry (New York),81(3),283-287.doi:10.1080/0033274 7.2018.1443676*” from the review. However, we cited it in “Discussion” section P33. We have included the following study: “*Amsalem, D., Hasson-Ohayon, I., Gothelf, D., & Roe, D. (2018). Subtle ways of stigmatization among professionals: The subjective experience of consumers and their family members. Psychiatric Rehabilitation Journal, 41(3), 163-168.doi:10.1037/prj0000310*”.  In “Results” section and in “Fig 1. PRISMA flowchart” We have stated that “two potential studies were identified by a subject matter expert” | P12 line 251  P14 Table 1 |
| I understand that the article followed rigorous strategies with regard to the meta-synthesis approach, but I think that some limitations of it should be acknowledged. The authors did not, as I understand it, get access to the original data transcripts, so their ability to identifying cross-cutting themes is restricted to what was already reported in the published studies. This may mean that the identification of new themes can’t truly be separated from what was identified by the original study authors, and should be noted as a limitation, as one of the purposes of the study was to identify “cross-cutting” themes. | Thank you for pointing this out. We agree that this is a limitation of most reviews using a meta-synthesis approach. We have modified the limitations section accordingly. | P36 line 764 |
| Something else that I wonder if could be brought in in the discussion section is the way in which expectations for the care of the “incompetent,” which has been identified as a factor that might increase social acceptance and decrease stigma within Muslim communities (Youssef, H. A., & Youssef, F. A. (1996). Evidence for the existence of schizophrenia in medieval Islamic society. History of Psychiatry, 7, 55– 62.) might also increase burden among family members. | Thank you for the suggestion. We have amended the discussion section to mention this point but it was not supported by the findings of the current review. | P34 line 714 |

Thank you for the given comments and constructive feedback. We hope the revised version is now suitable for publication and looking forward to hearing from you in due course.

Sincerely,

Aisha Alyafei

*PhD student*

*UCL Division of Psychiatry*
